# A Comprehensive Review of Non-Invasive Core Body Temperature Measurement Techniques

**DOI:** 10.3390/s26030972

**Published:** 2026-02-02

**Authors:** Yuki Hashimoto

**Affiliations:** Department of Mechanical Engineering, Institute of Science Tokyo, Meguro-ku, Tokyo 152-8552, Japan; hashimoto.y.e45a@m.isct.ac.jp

**Keywords:** core body temperature, non-invasive measurement, wearable sensors, in-ear thermometry, infrared thermography, ingestible sensors, heat-flux sensing

## Abstract

Core body temperature (CBT) is a fundamental physiological parameter tightly regulated by thermoregulatory mechanisms and is critically important for heat stress assessment, clinical management, and circadian rhythm research. Although invasive measurements such as pulmonary artery, esophageal, and rectal temperatures provide high accuracy, their practical use is limited by invasiveness, discomfort, and restricted feasibility for continuous monitoring in daily-life or field environments. Consequently, extensive efforts have been devoted to developing non-invasive CBT measurement and estimation techniques. This review provides an application-oriented synthesis of invasive reference methods and representative non-invasive approaches, including in-ear sensors, infrared thermography, ingestible telemetric sensors, heat-flux-based techniques, and model-based estimation using wearable physiological signals. For each approach, measurement principles, accuracy, invasiveness, usability, and application domains are comparatively examined, with particular emphasis on trade-offs between measurement fidelity and real-world implementability. Rather than ranking methods by absolute performance, this review highlights their relative positioning across clinical, occupational, and daily-life contexts. While no single non-invasive technique can universally replace invasive gold standards, recent advances in wearable sensing, heat-flux modeling, and multimodal estimation demonstrate growing potential for practical CBT monitoring. Overall, the findings suggest that future CBT assessment will increasingly rely on hybrid and context-aware systems that integrate complementary methods to enable reliable monitoring under real-world conditions. This review is intended for researchers and practitioners who need to select or design CBT monitoring systems.

## 1. Introduction

Core body temperature (CBT) is a fundamental physiological parameter that represents the temperature of deep tissues and organs within the body, including the viscera, blood, and central nervous system, and is distinct from surface skin temperature. CBT is tightly regulated by the thermoregulatory system centered in the hypothalamus and is typically maintained within a narrow range of approximately 36.5–37.5 °C at rest, while dynamically changing in response to physical activity, environmental temperature, and circadian rhythms [1,2]. In contrast to skin temperature, which is strongly influenced by ambient conditions, clothing, and peripheral blood flow and does not necessarily reflect internal physiological states, CBT directly represents the internal thermal environment resulting from metabolic heat production, circulatory dynamics, and sweating responses. Consequently, CBT plays a critical role in heat stress assessment, maintenance of organ function, perioperative hypothermia management during anesthesia, and diagnosis of infectious and febrile diseases [3]. In addition, CBT is widely recognized as a key physiological indicator in sleep and circadian rhythm research [2].

Despite its physiological importance, accurate measurement of CBT remains challenging. According to its strict definition, reliable assessment requires temperature measurements at sites close to the central circulation, such as the pulmonary artery, esophagus, rectum, or urinary bladder. However, these approaches are invasive or semi-invasive and impose substantial limitations in terms of wearability, user comfort, and feasibility for long-term or free-living measurements [4]. In clinical and experimental settings, esophageal and rectal temperatures have long been regarded as gold standards for CBT measurement; nevertheless, issues such as discomfort during probe insertion, risk of infection, and practical difficulties during exercise or occupational activities persist [5]. These limitations have driven growing global interest in the development of non-invasive techniques for daily and continuous CBT monitoring.

Representative non-invasive approaches include in-ear devices that measure temperature near the external auditory canal [6], infrared thermography for acquiring and analyzing body surface temperature distributions [7], ingestible telemetric pill-type sensors [8], heat-flux-based methods relying on skin temperature and heat-flux measurements [9], and algorithmic estimation techniques that estimate CBT from other physiological signals such as heart rate, skin temperature, and heat flux [10]. Each of these methods exhibits distinct advantages and limitations with respect to accuracy, temporal responsiveness, wearability, and robustness to environmental influences. Therefore, clarifying the technical characteristics and application constraints of existing CBT monitoring approaches is essential to guide subsequent methodological comparison and system design. In this review, we specifically focus on technologies aimed at continuous and non-invasive estimation of core body temperature, rather than conventional spot measurements such as oral or axillary thermometry, which primarily provide surrogate surface temperature readings and fall outside the main scope of this work.

As summarized in Table 1, most previous review papers have focused on a limited subset of CBT sensing techniques rather than providing a comprehensive overview [4,8,11,12,13,14,15,16,17]. In contrast, this review covers a wide range of CBT measurement and estimation approaches, including the latest studies, and offers an application-oriented comparison by explicitly addressing the trade-offs among accuracy, invasiveness, and real-world usability. Specifically, both invasive and non-invasive techniques—such as in-ear sensors, infrared thermography, ingestible sensors, heat-flux-based methods, and model-based estimation approaches—are reviewed, and their applicability in clinical, occupational, and daily-life contexts is discussed. This review is intended for researchers and practitioners seeking to select or design CBT monitoring systems tailored to specific application requirements.

## 2. Core Body Temperature Measurement Techniques

### 2.1. Invasive and Semi-Invasive Reference Measurements

The most reliable approaches for evaluating CBT involve invasive measurements in which temperature probes are inserted into anatomical sites located close to the central circulation, enabling direct assessment of internal temperature. Representative measurement sites include the esophagus, rectum, urinary bladder, and pulmonary artery. Because these sites are adjacent to deep trunk blood flow and are relatively insulated from ambient temperature and fluctuations in skin blood perfusion, they are considered to provide measurements closest to the “true” CBT [18]. Among them, pulmonary artery blood temperature directly reflects the temperature of the central circulation and has long been regarded as the reference standard in intensive care units and operating rooms [19]. However, pulmonary artery catheterization is a highly invasive medical procedure associated with serious risks, including vascular injury, arrhythmias, and infection, making it impractical for routine physiological research or use in daily-life settings [20].

Esophageal temperature is measured by inserting a temperature probe into the thoracic esophagus, which lies in close proximity to the heart and major blood vessels, and is widely used in anesthetized surgical procedures and exercise physiology studies [21]. Esophageal temperature exhibits relatively rapid responsiveness to thermal perturbations and can sensitively capture CBT changes during exercise or conditions involving pronounced thermoregulatory responses [22]. Nevertheless, probe insertion causes discomfort to participants, and measurements are susceptible to artifacts arising from swallowing and body movement, rendering this method unsuitable for continuous monitoring in daily life [5].

Rectal temperature has been the most widely used surrogate of CBT and has served as a reference measure across clinical medicine, exercise science, and environmental physiology [23]. The rectum is well perfused and exhibits relatively small temperature fluctuations, resulting in high measurement stability. However, rectal temperature responds more slowly to changes in CBT than esophageal temperature, and during intense exercise it may lag behind increases in CBT by several minutes [24]. In addition, issues related to privacy, hygiene, and user acceptance substantially limit its utility for long-term or field-based measurements, making long-term or field-based measurements impractical [25,26].

Bladder temperature is measured using a temperature sensor integrated into a urinary catheter. Because urine in the bladder is thermally coupled to deep abdominal blood flow, bladder temperature is often used as an indicator of CBT in postoperative care and intensive care settings [27]. However, its accuracy may be influenced by urine volume, and catheter insertion carries an inherent risk of infection, imposing clinical constraints that limit its general applicability [28].

In summary, invasive and semi-invasive measurement techniques—including pulmonary artery, esophageal, rectal, and bladder temperatures—provide the most accurate assessment of CBT and are widely regarded as reference standards. Nevertheless, due to discomfort during probe insertion, restrictions on movement, hygiene requirements, and infection risks, these methods are unsuitable for continuous, long-term, or everyday monitoring. Consequently, their use is largely confined to controlled environments such as operating rooms, intensive care units, laboratories, or rigorously supervised exercise physiology studies. At the same time, these reference measurements play a central role in the validation and benchmarking of non-invasive CBT estimation methods, including wearable in-ear devices, heat-flux-based techniques, and machine-learning-based models, and remain indispensable for the development and evaluation of emerging CBT monitoring technologies.

### 2.2. Non-Invasive and Minimally Burdensome Measurement Approaches

Non-invasive and minimally burdensome approaches for CBT monitoring have been actively investigated to overcome the practical limitations of invasive reference measurements. In this review, “non-invasive” refers to techniques that do not involve skin penetration or insertion into body cavities, whereas “minimally invasive” denotes methods that require only minor physiological intervention without tissue injury. Ingestible telemetric sensors occupy an intermediate position between these two categories, as they require oral ingestion but do not involve invasive tissue penetration.

Based on this classification, the approaches discussed in this section include in-ear temperature sensors, infrared thermography, ingestible telemetric sensors, heat-flux-based methods, and model-based estimation techniques using wearable physiological and environmental signals. These methods differ substantially in their measurement principles, achievable accuracy, wearability, and robustness to environmental and physiological variability.

Accordingly, ingestible sensors are treated as quasi-non-invasive approaches in this review. Although they do not require probe insertion through the skin or body cavities, they involve oral administration and are therefore not entirely intervention-free, which should be considered when interpreting their applicability.

#### 2.2.1. In-Ear Temperature Sensors for Core Body Temperature Monitoring

The ear is located close to the brain and major blood vessels such as the carotid arteries, and tympanic membrane temperature has been shown to closely reflect changes in deep brain temperature [29]. For this reason, infrared tympanic thermometers that measure eardrum temperature have long been widely used in clinical and non-clinical settings [30]. However, infrared tympanic thermometers are designed for brief, spot measurements and are therefore unsuitable for continuous monitoring [31]. Moreover, maintaining long-term contact between a sensor and the tympanic membrane is impractical and uncomfortable for users [32].

To address these limitations, wearable in-ear devices that measure temperature near the entrance of the ear canal have been actively investigated [6,33,34,35,36,37,38]. For example, Ota et al. developed a wearable device equipped with an infrared sensor for measuring ear canal temperature with the aim of enabling daily CBT monitoring, and demonstrated good agreement between the developed device and a commercially available ear thermometer under hot and exercise conditions [6]. Chaglla et al. reported improved temperature measurement accuracy by coating the surface of a commercial infrared sensor with graphene ink [33]. However, these studies were conducted with a limited number of participants under relatively idealized exercise conditions, and they did not employ invasive gold-standard reference measurements, as described in Section 2.1, but instead relied on commercial ear thermometers. Therefore, the agreement between CBT and device-measured temperatures should be interpreted with caution.

Ko et al. investigated the relationship between ear canal temperature measured using an infrared sensor and rectal temperature during sleep in female participants [34]. Their results showed that a sufficient insertion depth (approximately 14 mm) was required to achieve a strong correlation between ear canal and rectal temperatures. This finding reflects the presence of a temperature gradient from the tympanic membrane to the ear canal entrance, caused by environmental influences, resulting in position-dependent temperature differences. Roossien et al. evaluated the feasibility of using a commercial wearable ear-mounted sensor for heat stress management in workers exposed to high thermal loads [35]. Although a high correlation was observed between the wearable sensor readings and hospital-grade infrared thermometers, the authors reported that environmental changes and personal protective equipment adversely affected measurement accuracy and occasionally caused abnormal readings, highlighting challenges in highly variable environments.

Kato et al. reported a reliability assessment of a wearable infrared ear canal thermometer (VTB01, Vitarate, Tokyo, Japan) designed for sports and occupational applications [36]. They evaluated measurement accuracy under various hot and exercise conditions while periodically introducing airflow toward the participants. Although systematic errors attributable to environmental changes were observed, the accuracy remained within acceptable limits under the tested conditions. However, the authors noted that more severe heat stress, exercise intensity, or environmental variability could further increase measurement errors. In addition, the use of medical film to reduce heat exchange between the ear canal and the external environment likely mitigated environmental effects, and performance without such insulation may be lower. To improve robustness against environmental variability, Olson et al. developed a device incorporating two temperature sensors positioned at different locations within the ear canal [31]. Their results demonstrated improved accuracy compared with a single-sensor configuration when evaluating errors associated with ambient temperature differences and inter-individual CBT variation. Nevertheless, this study did not consider time-varying ambient temperature or airflow effects, which are commonly encountered in real-world use scenarios. Zhang et al. reported an in-ear device featuring a longer insertion depth (27 mm) designed to measure temperature closer to the tympanic membrane for heat-stroke prevention applications [37]. When properly positioned, the device showed good agreement with rectal temperature during exercise in hot environments, except immediately after the onset and cessation of exercise. However, the study involved a small sample size (six participants) and was conducted under controlled laboratory conditions.

Overall, in-ear temperature sensors offer reduced user burden compared with gold-standard invasive CBT measurements, as they require only device placement in the ear. However, because the ear canal is inherently susceptible to external environmental influences, achieving robust measurements under dynamic conditions remains a key challenge. Although potential applications include sports, occupational settings, and fever screening—where substantial environmental variation and CBT elevation are expected [5,31,35,36,37]—current evidence of effectiveness is largely limited to sleep monitoring [27], intraoperative settings [38], and light activities performed under relatively stable environmental conditions [35]. While several strategies to enhance robustness against environmental disturbances have been proposed [31,37], validation has so far been confined to idealized conditions, underscoring the need for reliability assessments that better reflect real-world usage scenarios.

#### 2.2.2. Infrared Thermography for Non-Contact Temperature Assessment

Infrared thermography (IRT) is a non-contact and non-invasive temperature measurement technique that estimates surface temperature by detecting infrared radiation emitted from the human body [7]. All objects with temperatures above absolute zero emit infrared energy as a function of their temperature. IRT passively detects mid- to long-wavelength infrared radiation emitted from the skin and converts the measured radiative intensity into an apparent temperature using the Stefan–Boltzmann law, enabling acquisition of high-spatial-resolution maps of skin temperature distribution [7]. Unlike conventional contact-based thermometers, IRT does not require physical contact between the subject and the measurement device. As a result, it has been widely applied and investigated in a broad range of contexts, including fever screening during infectious disease outbreaks [7], as well as medical, industrial, and animal health management applications [39,40].

IRT offers several notable advantages for body temperature assessment. The primary advantage is its non-invasive nature, as temperature can be measured without direct contact, thereby minimizing discomfort and reducing the risk of cross-contamination [41]. This characteristic is particularly valuable in situations where conventional temperature measurement methods are difficult to apply, such as mass screening of large populations or monitoring of vulnerable groups. During the COVID-19 pandemic, non-contact infrared cameras and so-called “forehead thermometers” were widely deployed at various checkpoints to rapidly identify individuals with suspected fever while maintaining physical distancing [42]. In addition, the ability to measure temperature remotely is important for minimizing stress on subjects [41,43]. In veterinary medicine and pediatric care, rectal thermometry has long been considered the standard for core temperature assessment; however, it imposes substantial stress on animals and infants and is often impractical for repeated measurements in large populations. In this context, IRT has attracted attention as a simple and animal-welfare-conscious approach, enabling frequent or continuous temperature monitoring without physical restraint [41,43]. For example, in livestock health management, measuring temperatures around the eye region or across the body surface using IRT has been explored as a means to detect signs of fever or inflammation without excessive handling of animals [43].

Another important advantage of IRT is its rapid measurement capability and broad spatial coverage [44]. Modern infrared cameras can scan an individual almost instantaneously and can continuously screen multiple individuals per minute, making them particularly useful in high-traffic environments such as airports, hospitals, and disaster triage centers [44]. In emergency and disaster response scenarios, thermal imaging cameras enable rapid detection of human heat signatures even in darkness or smoke, and are routinely used by firefighters and rescue teams to locate survivors and identify thermal hotspots [45]. Furthermore, thermographic images provide full-body surface temperature maps, allowing spatial analysis of temperature distributions [44]. This capability enables visualization of localized thermal abnormalities, such as regions of inflammation or infection and heat distribution during physical activity, which may be missed by single-point temperature measurements [45]. In occupational health and sports medicine, for example, visualizing how different body regions heat up during work or exercise can help identify areas exposed to excessive thermal load and support the development of effective cooling strategies [45,46]. Overall, owing to its non-contact and real-time characteristics, IRT represents a highly convenient and versatile technology for temperature assessment in both humans and animals.

Despite these advantages, important limitations exist when IRT is used for estimating CBT, particularly with respect to accuracy and reliability [14,16,47]. Skin temperature serves only as an indirect surrogate of CBT, and the relationship between the two can vary substantially depending on both physiological and environmental factors [16,23,47]. Under stable indoor conditions and with appropriate measurement protocols, thermographic temperatures measured at specific anatomical sites—such as the inner canthus of the eye—have been shown to reasonably track internal temperature [48,49,50]. However, in practice, the sensitivity and specificity of IRT for fever screening are often insufficient. Numerous studies have reported high false-negative rates, in which non-contact infrared devices fail to detect individuals who are truly febrile [50]. For example, historical reports have documented extremely low detection rates of infected individuals during large-scale airport screening using thermal imaging systems [50]. During the 2014 Ebola virus disease outbreak and the SARS epidemic, airport-based IRT screening failed to identify any confirmed cases, corresponding to a sensitivity of 0% in some instances [51]. Similarly, during the early phase of the COVID-19 pandemic, only a few percent of cases worldwide were detected through airport temperature screening [52,53]. While some of these failures can be attributed to improper deployment or operational issues, they also stem from the inherent physiological variability of skin temperature [14,47,54,55]. Factors such as ambient temperature, airflow, recent physical activity, sweating state, and inter-individual metabolic differences can influence skin temperature independently of CBT [14,47]. The forehead is a common measurement site for handheld infrared thermometers; however, it is highly sensitive to environmental cooling and heating, and immediately after exposure to hot or cold environments, forehead temperature often fails to accurately reflect internal thermal status [16,55,56]. Indeed, simple forehead scanning has been shown to be unable to track CBT changes during exercise or under heat stress [23,57,58,59,60,61]. For instance, studies have demonstrated that standard infrared thermometers failed to detect clinically elevated CBT in marathon runners or firefighters wearing protective clothing [57,60].

In response to these challenges, research efforts have focused on improving the robustness of IRT-based temperature assessment against environmental variability and inter-individual differences. Wang et al. proposed a sensing system combining IRT with auxiliary sensors, including an anemometer and temperature–humidity sensors, for applications in cattle temperature management, and reported that the combined system achieved improved CBT estimation accuracy compared with IRT alone in field experiments [62]. However, their validation was limited to indoor livestock facilities, and the effectiveness of the system in open or free-ranging environments remains unverified. Shan et al. developed a CBT prediction model for human temperature management in medical, sports, and elderly care contexts by integrating personal characteristics (e.g., sex, age, and ethnicity) with skin temperatures measured at multiple facial regions using IRT, reporting superior accuracy and consistency compared with conventional linear regression and machine-learning models [63]. Nevertheless, this model was validated using publicly available datasets collected under constrained indoor conditions, and its performance under extreme thermal environments or in the presence of physiological responses such as sweating has not yet been examined.

In summary, although IRT-based CBT monitoring has been explored across a wide range of applications owing to its high convenience and non-contact nature, raw infrared measurements alone do not constitute a complete substitute for direct CBT assessment. Environmental sensitivity, physiological variability, and user- and device-related errors collectively limit the ability of IRT to provide accurate internal temperature estimates under diverse conditions. At present, IRT does not deliver precise internal temperature measurements across all scenarios and, at best, functions as a screening tool for identifying individuals with suspected fever. Nevertheless, ongoing efforts to integrate IRT with other sensors and machine-learning techniques may help overcome these limitations, potentially enabling broader and more reliable applications in the future.

#### 2.2.3. Ingestible Telemetric Sensors for Core Body Temperature Measurement

Ingestible sensors, also referred to as smart pills, are non-invasive monitoring technologies that are orally administered, introduced into the gastrointestinal (GI) tract, and capable of measuring physiological signals from within the body and wirelessly transmitting the data to an external receiver [64,65,66]. At present, GI temperature measurement using ingestible sensors is widely employed as an alternative approach for CBT assessment, replacing invasive measurements such as rectal or esophageal temperature monitoring [64]. The operating principle of ingestible temperature sensors is relatively straightforward: a miniature capsule containing a temperature-dependent sensing element, such as a thermistor or quartz crystal oscillator, is swallowed, and temperature data measured within the GI tract are transmitted wirelessly to an external receiver [64]. Early concepts of ingestible temperature sensing were proposed in the 1980s [67,68], and since the 2000s, advances in wireless communication and miniaturization of electronic components have led to the commercialization of devices such as VitalSense^®^ (Philips Respironics, Murrysville, PA, USA) [69], CorTemp^®^ (HQ, Inc., Lake Worth, FL, USA) [70], myTemp^®^ (myTemp B.V., Nijmegen, The Netherlands) [71], and e-Celsius^®^ (BodyCap, Hérouville-Saint-Clair, France) [72]. These technologies have been increasingly applied in exercise physiology, clinical medicine, and occupational safety research [8].

The primary advantage of ingestible sensors for CBT monitoring lies in their ability to provide non-invasive and continuous measurements under free-living conditions [8]. Although rectal and esophageal temperatures are regarded as gold-standard indicators of CBT and offer high measurement reliability, they are invasive and often associated with substantial discomfort, limiting their practicality for long-term monitoring and field applications [8]. In contrast, once ingested, telemetric sensors can continuously record CBT for several hours to several tens of hours with minimal interference to daily activities or physical exercise [73]. This capability has made ingestible sensors a standard measurement tool in thermal and exercise physiology for recording CBT responses to heat stress and physical exertion, as well as for assessing the risk of heat-related illnesses such as heat stroke [70,74,75,76]. Successful applications have also been reported in demanding environments where conventional methods are impractical, including among divers, long-distance runners, military training scenarios, soccer players, and triathletes [71]. In clinical contexts, ingestible sensors have been investigated for fever monitoring, with studies reporting that GI temperature measurements provide higher reliability than axillary or non-contact forehead temperature measurements. Such approaches may facilitate improved characterization of fever patterns, earlier detection of conditions such as sepsis, and reduced workload for nursing staff by enabling automated and continuous temperature monitoring [77]. In veterinary and animal research, studies involving dogs have demonstrated that ingestible sensors can safely and reliably measure CBT, offering a temperature monitoring method that aligns with animal welfare considerations [78].

Despite these advantages, ingestible sensors present several important limitations. First, sensor movement within the GI tract over time and the influence of food and fluid intake can induce transient temperature drops or measurement bias, particularly during the early post-ingestion period or while the sensor remains in the stomach [79,80]. Consequently, many studies recommend initiating data analysis only after several hours have elapsed following ingestion to ensure measurement stability [80,81]. However, excessive delay increases the risk of sensor excretion, necessitating careful timing in both research and practical applications [78]. Second, systematic sensor-to-sensor bias and inter-device variability have been reported, requiring individual calibration or correction for high-precision research applications [8]. For example, Hunt et al. evaluated 119 commercially available ingestible sensors (myTemp^®^) in a temperature-controlled water bath and found that 59.7% exhibited bias exceeding specified accuracy thresholds, underscoring the need for pre-calibration within physiologically relevant temperature ranges [82]. Product-dependent differences have also been documented. Bongers et al. tested four commercial ingestible sensors (CorTemp^®^, e-Celsius^®^, myTemp^®^, and VitalSense^®^) in a water bath and reported that although all devices demonstrated high overall accuracy, notable differences in bias and response time existed among products [83]. Third, because ingestible sensors are excreted from the body within several tens of hours [8], long-term CBT monitoring over weeks or longer requires repeated ingestion. Excretion time varies considerably among individuals; for instance, Monnard et al. reported a mean excretion time of 31 h, ranging from 13 to 82 h after ingestion [73]. Therefore, prolonged monitoring necessitates individualized planning of ingestion schedules. Additional practical considerations include the predominantly disposable nature of current capsules, associated costs, and restrictions on use during magnetic resonance imaging (MRI) examinations [77].

Recent research trends have focused on further miniaturization and functional enhancement of ingestible sensors, alongside increased emphasis on power supply and safety considerations. Alternative energy strategies, such as energy harvesting from the GI environment and the use of biodegradable power sources, have been proposed to replace conventional button batteries, enabling safer and more sustainable designs [64,84]. Moreover, efforts toward multifunctional sensing capabilities and the development of mechanisms that allow prolonged residence within the GI tract are ongoing [65]. These advances are expected to further enhance the functionality, usability, and application scope of ingestible telemetric sensors for CBT monitoring.

#### 2.2.4. Heat-Flux-Based Methods for Core Body Temperature Estimation

Methods that estimate CBT by measuring temperature and heat flux on the skin surface have been investigated for several decades as non-invasive alternatives to invasive gold standards such as rectal and esophageal temperatures [9,85,86,87]. Representative approaches within this framework include the zero-heat-flux (ZHF) method [85,86,88,89,90,91,92,93], the single-heat-flux (SHF) method [9,94,95,96,97,98,99,100,101,102,103,104,105,106,107,108,109,110,111], and the dual-heat-flux (DHF) method [87,112,113,114,115,116,117,118,119,120,121]. All of these techniques share the common principle of estimating CBT based on thermal transport information obtained from sensors attached to the skin surface.

The ZHF method aims to actively eliminate heat flux between the skin and the environment, thereby driving the skin surface temperature toward equivalence with CBT [86]. This is achieved by placing multiple temperature sensors or a heat-flux sensor, combined with a heating element, on the skin surface and controlling the heating so that no net heat flow occurs across the skin boundary, establishing thermal equilibrium between the skin surface and deeper tissues. Based on this principle, ZHF-based devices have been explored for applications such as temperature management during cardiac and vascular surgery, as well as in hospital wards and intensive care units [88,89,90,91,92]. Representative commercial implementations of the ZHF principle, such as the 3M™ Bair Hugger™ temperature monitoring system (3M, Maplewood, MN, USA), further demonstrate the clinical maturity and reliability of this approach in perioperative and intensive-care settings. More recently, Lu et al. investigated the feasibility of ZHF methods for heat stress management under high-temperature environments and reported that an improved ZHF probe incorporating a Peltier element enabled stable CBT measurements even under hot conditions [93], extending potential applications beyond clinical settings. However, due to the need for active thermal control to maintain zero heat flux, ZHF systems typically require high power consumption and relatively thick probe structures, resulting in limited flexibility and wearability. These characteristics restrict their suitability for free movement and long-term portable monitoring, posing significant challenges for wearable applications [87,117]. In addition, reduced accuracy has been reported at CBT values below 32 °C [91], indicating limitations for hypothermic conditions.

The SHF method estimates CBT by simultaneously measuring skin temperature and heat flux and approximating the human body as a one-dimensional heat conduction model [9]. Unlike ZHF methods, SHF does not require active heating, enabling low-power operation and simpler device structures [117]. Consequently, SHF-based approaches have been widely investigated for applications such as circadian rhythm monitoring [94], exercise physiology, and heat stress monitoring in occupational safety contexts [9,94,95,102], making them attractive candidates for wearable CBT estimation during daily activities. In recent years, commercial devices such as CORE^®^ and Calera Research^®^, developed by greenteg AG (Rümlang, Switzerland), have become available, and studies have reported validation results for their use during exercise in hot environments and in bedside clinical applications [103,104,105,106,107,108,109,110,111]. However, several practical challenges remain. SHF methods typically require prior calibration against a reference measurement to account for inter-individual differences in thermal resistance of the skin and subcutaneous tissues [117]. Moreover, estimation accuracy is sensitive to environmental factors such as ambient temperature and airflow [92], and even slight changes in sensor–skin contact can disrupt heat-flux measurements and destabilize CBT estimates [113,114]. To address these limitations, recent studies have proposed self-calibration techniques that eliminate the need for reference measurements [98,100], as well as probe designs that enhance robustness against environmental disturbances [95,97,99,101]. Leveraging their inherent advantages of low power consumption and structural simplicity, SHF-based methods are expected to advance toward practical wearable devices as these challenges are progressively mitigated.

The DHF method extends the SHF approach by utilizing two heat-flux measurement paths with different thermal resistances, enabling more precise estimation of the temperature gradient between the skin surface and deeper tissues [87]. By simultaneously incorporating multiple heat-flux signals, DHF methods reduce uncertainty associated with skin-side thermal properties and have been reported to improve robustness against inter-individual variability [121,122]. Applications investigated to date include temperature management in clinical settings [116], circadian rhythm monitoring [116], and physiological condition assessment during work under hot environments [119,120]. Moreover, recent commercialization of a wearable dual-heat-flux sensor, Moni-Patch^®^ (Murata Manufacturing, Yokohama, Japan), indicates substantial progress toward practical deployment of DHF-based CBT monitoring technologies. Nevertheless, several fundamental challenges must be addressed for wearable implementation. First, because DHF systems rely on multi-layer or multi-channel structures, changes in skin contact can disrupt the ratio between heat-flux channels, leading to unstable estimates. Motion artifacts arising from walking, arm movement, and sweat-induced interface changes are difficult to avoid in real-world conditions and represent major sources of error [99,102,123]. Second, variations in skin blood flow and sweating during exercise alter local thermal resistance, violating the steady-state assumptions of the underlying thermal model and resulting in response delays and estimation errors [119,122]. Third, the additional thermal resistance layers, thermocouples, and wiring required for dual-channel configurations tend to increase sensor thickness and stiffness, reducing conformity to curved skin surfaces and compromising wearing comfort [121]. Furthermore, achieving complete robustness against ambient temperature fluctuations and airflow disturbances remains challenging, as small variations in heat flux can significantly affect estimation accuracy [102,123]. Addressing these issues will require advances in flexible and thin material design, interface stabilization mechanisms, compensation for environmental disturbances, and algorithmic corrections capable of handling dynamic physiological conditions.

A common advantage of heat-flux-based methods is their ability to provide continuous CBT monitoring through simple attachment to the skin surface without the need for invasive procedures. Unlike ingestible sensors, these methods are not constrained by limited usage duration or sensor migration within the body, enabling long-term and repeated measurements. Compared with IRT, heat-flux-based approaches are not limited by field-of-view constraints and can provide stable data acquisition during physical activity. However, each method exhibits distinct trade-offs. ZHF methods offer high accuracy and are well established in clinical practice but are constrained by power consumption and bulky probe structures that limit wearability. SHF methods are structurally simple and energy efficient, making them well suited for wearable applications, yet they remain sensitive to variations in tissue properties and environmental disturbances, which pose challenges for stable CBT estimation in daily-life settings. DHF methods improve robustness against tissue property uncertainty and external disturbances compared with SHF approaches but introduce increased structural complexity and reduced wearing comfort due to thicker and less flexible sensor designs. Overcoming these practical challenges in each method is expected to play a key role in expanding their applicable use scenarios in the future.

#### 2.2.5. Model-Based and Multimodal Estimation of Core Body Temperature

CBT is a critical indicator directly linked to the assessment of heat stress and excessive thermal load. However, direct measurements such as rectal or esophageal temperatures are invasive and operationally restrictive, making them unsuitable for continuous monitoring in occupational settings, sports, military operations, and daily-life environments [124,125,126]. To bridge this gap, a broad range of studies has explored the estimation of CBT from physiological and environmental signals that can be readily acquired using wearable devices, including heart rate (HR), skin temperature, heat flux, acceleration (activity level), and ambient conditions [10,25,124,125,126,127,128,129,130,131,132,133,134,135,136,137,138,139,140,141,142,143,144,145]. Among these inputs, HR has been widely adopted as a primary variable because it is closely linked to metabolic heat production and heat dissipation through circulatory regulation. Early frameworks proposed the use of Kalman filters and extended Kalman filters to perform sequential state estimation of CBT based on time-series observations [124,127]. More recently, multimodal estimation approaches incorporating skin temperature and/or heat flux—signals commonly used in heat-flux-based methods—have been investigated, demonstrating improved robustness and estimation performance compared with HR-only models [10,25,129,130,133,136,141,145].

The underlying principles of these approaches can be broadly categorized into physiology- and physics-based models and data-driven (statistical or machine-learning-based) models. In the former category, simplified compartmental or thermophysiological models that approximate human heat balance and heat transfer are employed to estimate CBT using wearable-derived inputs such as HR, skin temperature, activity level, and environmental conditions [129,135]. For example, Hamatani et al. combined wearable sensing with the Gagge two-node thermophysiological model, using HR, skin temperature, and ambient temperature and humidity acquired from wearable or portable sensors as inputs to compute the temporal evolution of CBT and skin temperature [128]. While such approaches offer strong physiological interpretability, they are constrained by parameter sensitivity and the inherent limitations of model simplification [135,145]. More recently, Hirata et al. developed a simplified compartment model derived from their previously reported high-resolution anatomical human model and combined it with wearable measurements to enable real-time CBT estimation, reflecting ongoing efforts to adapt physiologically grounded models for practical deployment [135].

In contrast, data-driven approaches learn a direct mapping from observed signals to CBT (or changes in CBT) using regression, state-space models, or machine-learning techniques, thereby improving adaptability to inter-individual and contextual variability [10,25,124,125,126,127,129,131,132,133,134,135,136,137,138,139,140,141,142,143,144]. In practice, hybrid approaches combining model-based and data-driven elements have also been reported. Kalman-filter-based frameworks, in particular, are well suited for real-time estimation under noisy field conditions because they allow recursive state updates with explicit handling of measurement uncertainty [130,145]. However, recent studies have highlighted that thermophysiological states during exercise (heating phase) and recovery (cooling phase) can differ substantially even at similar HR levels, leading to increased estimation errors when a single model is used. To address this issue, biphasic or multi-model approaches have been proposed to improve robustness while retaining HR-only estimation. For example, Rizvi and Falcone et al. introduced methods that switch between observation models for exercise and recovery phases based on HR [137,143]. Subsequently, Zhao et al. extended these approaches by dynamically updating the contribution of exercise and recovery observation models using weighted transitions or long short-term memory (LSTM) networks, enabling smoother and more adaptive model switching [144,145]. As a result, current research in this domain increasingly focuses on achieving a balance between computational efficiency suitable for wearable implementation and generalization performance across diverse conditions [126,137,143,144].

The advantages of model-based and multimodal CBT estimation approaches are threefold. First, they are entirely non-invasive and enable continuous data acquisition using existing wearable devices such as heart rate monitors and skin temperature sensors [10,124,140]. Second, they are well suited for environments where direct CBT measurement is impractical, including occupational heat safety management, physical activity under personal protective equipment (PPE), and heat risk monitoring in military, firefighting, and sports field settings [129]. In the context of occupational and industrial hygiene, CBT estimation models have been continuously evaluated and validated for assessing thermal load and managing heat stress risk in real-world workplaces [25,125,134,136,138,140,142]. Third, advances in signal processing and estimation algorithms have demonstrated that acceptable accuracy may be achieved even with single-input models based solely on HR, offering design flexibility to minimize sensor count and wearing burden [124,143].

At the same time, several limitations must be acknowledged. These approaches do not directly measure CBT; instead, they estimate it from proxy signals that can be influenced by non-thermal factors such as psychological stress, dehydration, cardiovascular adaptation, physical fitness, and circadian variation, making estimation robustness highly condition dependent [10,139,141]. For instance, studies estimating CBT from ECG-derived metrics (e.g., R–R intervals) have shown that input features are susceptible to diverse physiological fluctuations, highlighting the difficulty of absolute CBT estimation when inter-individual differences and diurnal variation are present [130]. Moreover, model validity may be constrained by environmental conditions (e.g., outdoor exposure, airflow, solar radiation) and subject characteristics, and caution is required when extrapolating laboratory-based validation results to real-world field settings [125,129,136,138]. Overall, rather than aiming to fully replace invasive CBT measurements, model-based and multimodal estimation approaches are increasingly evolving toward integrated frameworks that balance computational simplicity, robustness, and generalizability, combining model structure (e.g., biphasic or multi-model representations) with learning and adaptation mechanisms to enable practically useful CBT and heat-risk monitoring in real-world scenarios.

## 3. Discussion and Future Perspectives

**Figure 1:**
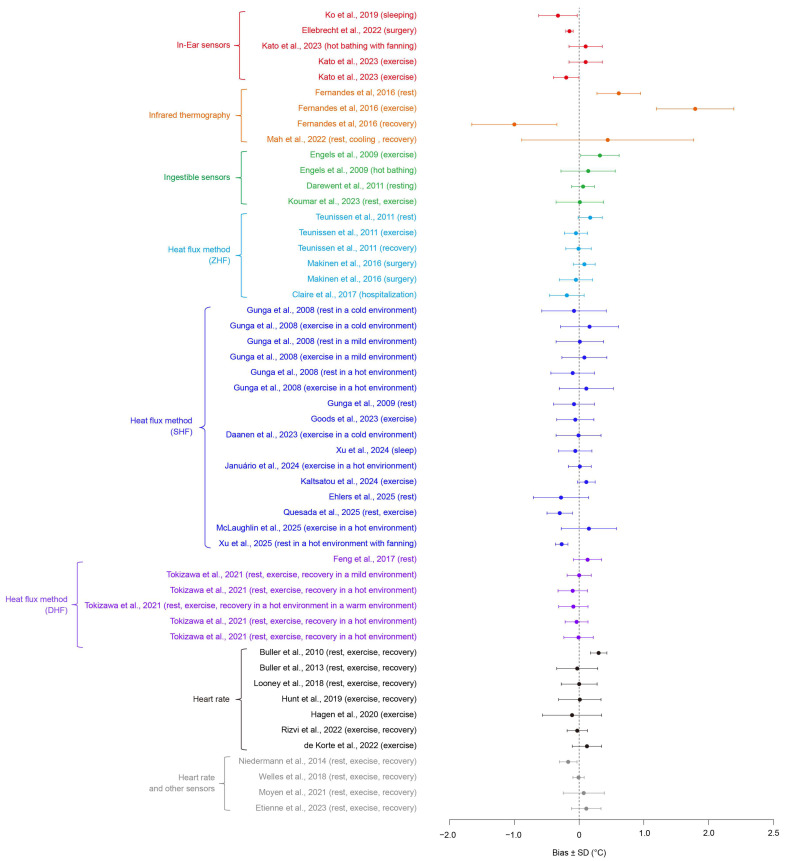
Summary of validation accuracy (Bias ± SD) reported in previous studies for CBT monitoring methods. For studies reporting 95% confidence intervals, the corresponding standard deviations were estimated by dividing the CI width by 1.96 [9,10,34,36,38,49,69,72,79,90,91,92,94,103,104,105,106,107,108,109,110,111,115,119,124,125,127,132,133,134,136,137,140,141,146].

Table 2 summarizes the advantages, limitations, and representative application scenarios of the various CBT measurement techniques reviewed in this paper. In addition, Figure 1 and Table 3 compile previously reported validation results regarding the accuracy of each method. Collectively, these comparisons highlight a fundamental design trade-off between measurement fidelity and practical implementability, reflecting intrinsic constraints imposed by sensing physics, human factors, and environmental variability. Consequently, the optimal configuration of CBT monitoring systems depends strongly on contextual factors, including target population, environmental conditions, and intended usage scenarios.

CBT is a physiologically well-defined parameter, and in situations where accurate determination of its absolute value is essential, invasive measurement methods remain indispensable. Temperatures measured at sites close to the central circulation, such as the pulmonary artery, esophagus, and rectum, have long been used as reference values (gold standards) for CBT assessment. However, these approaches impose substantial invasiveness and subject burden, which fundamentally limit their suitability for long-term or continuous monitoring in daily-life or field environments. Accordingly, invasive methods should be regarded as tools selectively employed in applications where maximum accuracy is prioritized, such as clinical medicine and fundamental physiological research.

Ingestible sensors occupy a unique position among CBT monitoring technologies, as they offer near-gold-standard accuracy while remaining non-invasive. Their ability to provide stable CBT measurements during exercise and under high-temperature conditions has led to widespread adoption in sports science, military research, and firefighting studies. Nevertheless, practical constraints—including single-use operation, limited measurement duration, cost, and irretrievability—restrict their applicability for routine, long-term daily monitoring. At present, ingestible sensors are therefore best positioned as reference tools for research and evaluation purposes. Looking ahead, advances that enable prolonged GI residence or controlled sensor positioning may substantially expand their potential use cases by facilitating longer-term monitoring.

In-ear sensors represent an intermediate solution in terms of CBT measurement accuracy among non-invasive, surface-based approaches. Their favorable wearability and capability for continuous monitoring make them well suited for use in daily life and occupational settings. However, measurement accuracy is limited by factors such as variability in sensor placement, inter-individual differences in ear canal anatomy, and susceptibility to ambient environmental conditions. As a result, in-ear sensors are more appropriate for tracking relative changes or identifying trends and risk levels, rather than for determining absolute CBT values with high precision.

IRT offers fully non-contact and rapid measurement and is among the most practical approaches from an implementation standpoint. It has therefore been widely deployed for fever screening during infectious disease outbreaks and for population-level assessment in public health contexts. However, its accuracy for estimating CBT at the individual level is limited and strongly influenced by environmental conditions and measurement sites. Accordingly, IRT should be positioned not as a quantitative CBT measurement technique, but rather as a screening tool specialized for identifying individuals with suspected fever.

Heat-flux-based sensors offer greater physiological relevance than methods relying solely on skin temperature, as they directly capture heat transfer near the skin. ZHF method provides high accuracy and has a strong track record in clinical applications, but its reliance on active heating mechanisms results in higher power consumption and thicker sensor structures, limiting its suitability for wearable deployment. SHF method enables simpler structures and low-power operation, making it attractive for wearables; however, it remains sensitive to variations in skin thermal properties and environmental disturbances. Recent advances in sensor design and signal processing have begun to mitigate these limitations. The DHF method further reduces uncertainty related to skin-side thermal properties by leveraging multiple heat-flux measurements, achieving a favorable balance between accuracy and practicality. Although system complexity remains a challenge, DHF currently appears to be one of the most promising approaches for next-generation wearable CBT sensors.

Heart-rate-based CBT estimation represents an approach with high practicality and moderate accuracy. While it does not directly measure CBT, its compatibility with existing wearable devices provides excellent scalability and extensibility. This makes it particularly suitable for early detection of increasing thermal load or transitions into hazardous heat stress zones. As such, HR-based estimation methods constitute practical options for occupational safety management, sports science, and daily health monitoring. Moreover, recent studies have demonstrated that fusing HR with additional signals such as skin temperature and heat flux can improve robustness against inter-individual variability and environmental changes, suggesting strong potential for further development as next-generation wearable CBT monitoring technologies.

In summary, the absence of a universally optimal CBT monitoring solution underscores the necessity of application-specific design strategies and adaptive system configurations. Accordingly, practical implementation should prioritize scenario-driven system integration, where complementary techniques are selectively combined to balance accuracy, usability, and robustness. Beyond improving individual techniques, future progress is likely to be driven by hybrid approaches that integrate complementary methods and by adaptive system designs that dynamically switch estimation strategies based on context and usage scenarios. Recent advances in integrated wearable physiological monitoring systems further highlight this trend; for example, Bao et al. developed an all-in-one smartphone-based intelligent photonic mHealth platform capable of simultaneous plantar, respiratory, and cardiac monitoring, demonstrating robust multimodal integration and clinical-grade performance [147]. Such developments are expected to further accelerate the real-world implementation and societal impact of CBT monitoring technologies.

## 4. Limitation of This Review

This review has several limitations. In particular, it was not conducted as a formal systematic review; rather, it aimed to provide a conceptual and application-driven synthesis of representative methods. Accordingly, the scope and coverage of the literature were guided by relevance to measurement principles, accuracy, invasiveness, and practical usability, rather than by predefined systematic inclusion criteria. In line with this scope, conventional clinical measurement sites such as oral and axillary temperatures were not included in the comparative tables and figures, as they primarily provide intermittent surrogate measurements and do not enable continuous CBT monitoring, which is the primary focus of this review.

Although this review does not follow a formal systematic review protocol, this choice was intentional. Given the substantial heterogeneity in sensing principles, experimental protocols, evaluation metrics, and application contexts among CBT monitoring technologies, a purely systematic synthesis would risk oversimplification and misleading comparisons. Instead, we adopted an application-oriented narrative review framework to provide structured technical insights and practical guidance for technology selection and system design.

In this review, literature was primarily identified using Google Scholar. Searches were performed using combinations of the keywords “core body temperature” or “body temperature” with terms related to invasive and non-invasive measurement techniques, including “esophageal temperature”, “rectal temperature”, “pulmonary artery temperature”, “in-ear temperature”, “infrared thermography”, “ingestible sensor”, “heat flux”, “zero-heat-flux”, “single-heat-flux”, “dual-heat-flux”, “heart rate”, “skin temperature”, “wearable”, “prediction”, and “estimation”. The search covered studies published mainly between 2000 and 2025, and additional relevant articles were identified by manually screening reference lists of key publications.

First, the present work primarily organized and compared CBT monitoring methods from the perspectives of measurement principles, accuracy, invasiveness, and practical implementability. However, in real-world applications, additional factors such as cost, maintainability, regulatory and certification requirements (e.g., medical device approval), and data privacy are also critical decision-making considerations. These aspects were not addressed in sufficient depth and remain important topics for future investigation.

Second, the discussion of accuracy and performance for each method relies on experimental conditions and evaluation metrics reported in the existing literature. The accuracy of CBT measurement and estimation can vary substantially depending on subject characteristics, environmental conditions, exercise modalities, and the definition of the reference temperature. Consequently, direct numerical comparison across different studies has inherent limitations. In this review, we therefore avoided making definitive claims regarding absolute superiority and instead focused on relative positioning and qualitative comparison among methods. Moreover, technical accuracy comparisons across studies are subject to inherent bias arising from inconsistencies in reference CBT measurement standards. While some studies adopted rectal temperature as the reference, others used esophageal, pulmonary artery, or bladder temperatures, each reflecting slightly different physiological compartments and response characteristics. Such heterogeneity may introduce systematic bias into cross-study comparisons of reported accuracy metrics. Future reviews could mitigate this limitation by adopting a reference-standard–stratified comparison framework, in which validation results are grouped and analyzed according to the specific CBT reference method employed. In addition, several validation studies of emerging CBT monitoring techniques were conducted with relatively small sample sizes, which limits the generalizability of their conclusions, particularly for free-living and long-term monitoring scenarios.

Third, many emerging approaches based on machine learning and sensor fusion are still at an early research stage, and their long-term stability and generalization performance in real-world environments have not yet been sufficiently validated. As a result, the future perspectives presented in this review reflect potential extensions of current technologies and should be re-evaluated as further empirical evidence becomes available through large-scale and long-term validation studies.

Fourth, available validation data for special populations, such as pediatric and elderly subjects or patients with chronic diseases, remain limited for many wearable CBT monitoring technologies. In particular, ethical constraints, compliance challenges, anatomical differences, and device size limitations restrict large-scale experimental investigations in pediatric cohorts. Consequently, systematic evaluation of population-specific performance and applicability is currently insufficient, and further dedicated studies are required to establish robust evidence for these groups.

## 5. Conclusions

This review summarized representative measurement and estimation approaches for CBT monitoring and compared their underlying principles, accuracy, invasiveness, usability, and application potential. Invasive reference techniques, non-invasive sensing approaches—including in-ear sensors, IRT, ingestible sensors, and heat-flux-based methods—as well as model-based estimation using heart rate and other wearable physiological signals were reviewed from the perspective of the trade-offs between measurement fidelity and practical implementability.

The comparison demonstrates that no single optimal solution exists for CBT monitoring across all application scenarios. Invasive techniques remain indispensable for providing accurate reference values but are inherently unsuitable for continuous monitoring in daily-life or field environments. Ingestible sensors enable non-invasive measurements with accuracy close to invasive standards; however, their use is constrained by limited measurement duration and operational considerations. In contrast, in-ear sensors, heat-flux-based sensors, and model-based estimation approaches offer superior wearability and deployability, albeit with inherent limitations in absolute accuracy, positioning them as practical solutions for real-world CBT assessment rather than strict replacements for gold-standard methods.

From an application-oriented perspective, different non-invasive CBT monitoring technologies exhibit distinct advantages across specific usage scenarios. Ingestible sensors are particularly well suited for academic research and high-intensity physical activities, such as sports science, military training, and firefighting studies, where high accuracy under dynamic thermal stress is required. In-ear sensors, SHF and DHF methods, and heart-rate-based estimation approaches provide favorable trade-offs between wearability, continuity, and usability, making them suitable for long-term daily monitoring and occupational safety applications. In contrast, IRT is best positioned as a rapid, non-contact screening tool for large-scale fever detection in clinical and public health contexts. The ZHF method, with its established clinical reliability, remains most appropriate for perioperative and critical-care monitoring.

Among non-invasive techniques, heat-flux-based approaches provide relatively high physiological relevance by directly capturing thermal transport near the skin. In particular, the DHF method shows promise in mitigating inter-individual variability and uncertainty in skin thermal properties. Meanwhile, CBT estimation based on heart rate and multimodal wearable signals offers high scalability by leveraging existing wearable platforms and continues to evolve as a practical tool for heat-risk detection and safety management, in addition to absolute CBT estimation.

Looking ahead, future progress in CBT monitoring will require not only incremental improvements in individual techniques but also the development of hybrid and context-aware systems that integrate complementary sensing modalities and dynamically adapt estimation strategies to application-specific contexts. Representative examples include the combination of in-ear sensors for continuous temperature tracking with heart-rate-based multimodal estimation to enhance environmental robustness in wearable applications, as well as clinical workflows integrating IRT for rapid fever screening with ingestible sensors for diagnostic verification.

Future research priorities can be broadly structured along three interconnected directions: (i) technology optimization, such as miniaturization, flexibility enhancement, and interface stabilization of wearable sensors, particularly for DHF-based systems; (ii) data fusion and algorithmic development, including real-time multimodal sensor fusion, adaptive modeling, and machine-learning-based compensation for inter-individual and environmental variability; and (iii) scenario-driven system implementation, exemplified by early-warning platforms for occupational heat stress, continuous health monitoring in daily life, and large-scale public health surveillance

In parallel, several unresolved challenges remain to be addressed, including long-term wearing comfort and skin irritation, secure handling and privacy protection of multimodal physiological data, and the development of low-cost, energy-efficient solutions suitable for deployment in resource-limited settings.

CBT remains a key physiological indicator across diverse domains, including heat illness prevention, occupational safety, sports science, and public health. The systematic organization and application-oriented perspective presented in this review are expected to provide a useful framework for future research, technological development, and the practical deployment of CBT monitoring systems.

## Figures and Tables

**Table 1:** Coverage of methods in each review.

Review (Year)	In-Ear Sensors	Ingestible Sensors	Infrared Thermography	Heat-Flux-Based Method			HR-Based Method	Mult-Sensor Fusion
				Zero-Heat-Flux Method	Single-Heat-Flux Method	Dual-Heat-Flux Method		
Moran et al. (2002) [11]	✓	—	—	—	—	—	—	—
Byrne et al. (2007) [8]	—	✓	—	—	—	—	—	—
Tamura et al. (2018) [4]	✓	—	✓	✓	—	✓	—	—
Conway et al. (2020) [12]	—	—	—	✓	—	—	—	—
Cutuli et al. (2021) [13]	✓	—	—	✓	—	—	—	—
Falcone et al. (2021) [14]	—	—	—	✓	✓	—	✓	✓
Foster et al. (2021) [15]	—	—	✓	—	—	—	—	—
Dolson et al. (2022) [16]	—	—	—	—	—	—	✓	✓
Zhao et al. (2023) [17]	—	—	✓	—	—	—	—	—
This study	✓	✓	✓	✓	✓	✓	✓	✓

Legend: ✓ = covered; — = not covered.

**Table 2:** Summary of CBT monitoring methods, including their advantages, limitations, and representative applications.

Method	Advantages	Limitations	Representative Application Scenarios
Invasive measurement (pulmonary artery, esophageal, rectal)	Highest accuracy Closest to physiological definition Gold standard	Highly invasive Low usability Unsuitable for long-term or field use	Surgery and anesthesia management Intensive care Physiological reference measurements
In-ear sensors	Non-invasive Good wearability Suitable for continuous monitoring Low-moderate cost	Sensitive to placement and ambient conditions Limited accuracy	Daily health monitoring Sports and occupational safety Wearable healthcare
IRT	Non-contact Rapid measurement Suitable for mass screening	Low individual accuracy Strongly affected by environment Indirect estimation	Fever screening during pandemics Public health surveillance Disaster response
Ingestible sensors	High accuracy close to invasive methods	Single-use Limited monitoring duration High cost (50–100 USD per use) Requiring FDA/CE medical certification Recovery issues	Sports science Military and firefighting training Validation studies
ZHF method	High accuracy Clinically validated Continuous monitoring	Requires active heating High power consumption Bulky sensor High cost (>500 USD)	Perioperative monitoring Intensive care Clinical research
SHF method	Simple structure Low power consumption Wearable potential	Sensitive to skin thermal properties and environment Limited robustness High cost (USD 325–1000)	Wearable prototypes Moderate-accuracy field monitoring
DHF method	Improved robustness to individual variability Better balance of accuracy and wearability	Increased complexity Calibration and motion artifacts High cost (USD 430)	Advanced wearable core temperature sensors Field and occupational monitoring
Heart rate–based estimation	Extremely high usability Uses existing wearables Low cost	Model dependency Training data required Generalization issues Strong individual dependence	Heat strain screening Large-scale population monitoring
Heart rate + other sensors (skin temperature, acceleration, etc.)	Higher accuracy than single-sensor methods Scalable Adaptive models	Model dependency Training data required Generalization issues	Industrial safety Sports monitoring Continuous daily-life assessment

**Table 3:** Summary of reported accuracy metrics for CBT monitoring methods.

Measurement Technique	Paper	Subject Types	Number of Validation Subjects, *n*	Environmental Conditions	Exercise Types	Reference for CBT	Bias ± SD [°C]
In-ear sensors	Ko et al. 2019 [34]	Healthy females (24 ± 3 years)	9	27 °C, 50% RH	Sleep	Rectal	−0.32 ± 0.30
	Ellebrecht et al. 2022 [38]	Make patients (72 ± 9 years)	10	N/A	Surgery	Bladder	−0.15 ± 0.06
	Kato et al. 2023 [36]	Healthy adults (23 ± 2 years)	9 (6 males, 3 females)	28 °C, 50% RH	Lower-limb warm-water immersion with 2 m/s airflow	Rectal	0.10 ± 0.26
	Kato et al. 2023 [36]	Healthy adult males (22 ± 1 years)	11	35 °C, 50% RH	Walking at 4 km/h with 0.5% slope	Rectal	0.10 ± 0.26
	Kato et al. 2023 [36]	Healthy adults (23 ± 3 years)	9 (5 males, 4 females)	35 °C, 65% RH	Running at 6 km/h with a 5% slope	Rectal	−0.20 ± 0.20
IRT	Fernandes et al. 2016 [49]	Healthy, physically active adult males (22.4 ± 3.3 years)	12	24.9 ± 0.6 °C, 62.3 ± 5.7% RH	Rest	Gastrointestinal	0.61 ± 0.34
	Fernandes et al. 2016 [49]	Healthy, physically active adult males (22.4 ± 3.3 years)	12	24.9 ± 0.6 °C, 62.3 ± 5.7% RH	Exercise (60% VO_2_max)	Gastrointestinal	1.79 ± 0.60
	Fernandes et al. 2016 [49]	Healthy, physically active adult males (22.4 ± 3.3 years)	12	24.9 ± 0.6 °C, 62.3 ± 5.7% RH	Recovery	Gastrointestinal	−1.00 ± 0.66
	Mah et al. 2022 [146]	Healthy adults (28.3 ± 9.4 years)	30 (14 males, 16 females)	23 °C, 55% RH	Rest, Lower-limb cold-water immersion, recovery	Oral	0.44 ± 1.33
Ingestible sensors	Engels et al. 2009 [69]	Healthy adult females (55.3 ± 5.9 years)	8	N/A	75% HRmax exercise	Rectal	0.32 ± 0.30
	Engels et al. 2009 [69]	Healthy adult females (55.3 ± 5.9 years)	8	N/A	40 °C whole-body warm-water immersion	Rectal	0.14 ± 0.42
	Darewent et al. 2011 [79]	Healthy adult males (22.4 ± 2.4 years)	11	21.0 ± 1.0 °C	Rest	Rectal	0.06 ± 0.18
	Koumar et al. 2023 [72]	Healthy adults (18–59 years)	23 (13 males, 10 females)	N/A	In hospital (24 h), fasting, light activity, normal sleep	Rectal	0.01 ± 0.37
Heat-flux method (ZHF)	Teunissen et al. 2011 [90]	Healthy moderately fit adults (28.3 ± 5.3 years)	7 (4 males, 3 females)	35 °C, 50% RH	Rest	Esophageal	0.17 ± 0.19
	Teunissen et al. 2011 [90]	Healthy moderately fit adults (28.3 ± 5.3 years)	7 (4 males, 3 females)	35 °C, 50% RH	Exercise	Esophageal	−0.05 ± 0.18
	Teunissen et al. 2011 [90]	Healthy moderately fit adults (28.3 ± 5.3 years)	7 (4 males, 3 females)	35 °C, 50% RH	Recovery	Esophageal	−0.01 ± 0.20
	Makinen et al. 2016 [91]	Vascular surgery patients (64 ± 13 years)	15 (11 males, 4 females)	N/A	Vascular surgery	Esophageal	0.08 ± 0.17
	Makinen et al. 2016 [91]	Cardiac surgery patients (68 ± 9 years)	15 (11 males, 4 females)	N/A	Cardiac surgery	Pulmonary artery	−0.05 ± 0.26
	Clare et al. 2017 [92]	ICU patients (54 ± 5 years)	52 (27 males, 25 females)	N/A	Rest	Esophageal	0.19 ± 0.27
Heat-flux method (SHF)	Gunga et al. 2008 [9]	Healthy adult males (39.5 ± 10.2 years)	20	10 °C	Rest	Rectal	−0.08 ± 0.50
	Gunga et al. 2008 [9]	Healthy adult males (39.5 ± 10.2 years)	20	10 °C	Exercise at 35%, 45%, and 55% VO_2_max)	Rectal	0.16 ± 0.45
	Gunga et al. 2008 [9]	Healthy adult males (39.5 ± 10.2 years)	20	25 °C	Rest	Rectal	0.01 ± 0.37
	Gunga et al. 2008 [9]	Healthy adult males (39.5 ± 10.2 years)	20	25 °C	Exercise at 25%, 45%, and 55% VO_2_max)	Rectal	0.08 ± 0.35
	Gunga et al. 2008 [9]	Healthy adult males (39.5 ± 10.2 years)	20	40 °C	Rest	Rectal	−0.10 ± 0.34
	Gunga et al. 2008 [9]	Healthy adult males (39.5 ± 10.2 years)	20	40 °C	Exercise at 25%, 35%, and 45% VO_2_max)	Rectal	0.11 ± 0.42
	Gunga et al. 2009 [94]	Healthy adult males (31.9 ± 8.0 years)	7	N/A	Rest	Rectal	−0.08 ± 0.32
	Goods et al. 2023 [103]	Athlete females (26 ± 4 years)	19	31.0 °C, 38.5% RH	Exercise (Training)	Gastrointestinal	−0.06 ± 0.29
	Goods et al. 2023 [103]	Athlete females (26 ± 4 years)	19	32.2 °C, 50% RH	Exercise (Match)	Gastrointestinal	−0.10 ± 0.37
	Goods et al. 2023 [103]	Athlete females (26 ± 4 years)	19	27.6 °C, 80% RH	Exercise (Match)	Gastrointestinal	−0.17 ± 0.42
	Goods et al. 2023 [103]	Athlete females (26 ± 4 years)	19	27.4 °C, 74% RH	Exercise (Match)	Gastrointestinal	−0.34 ± 0.32
	Daanen et al. 2023 [104]	Healthy adults (24.3 ± 1.2 years)	9 (4 males, 5 females)	18 °C, 50% RH	Rest, exercise (cycling), recovery	Rectal	−0.01 ± 0.35
	Xu et al. 2024 [105]	Healthy adults (20–25 years)	14 (7 males, 7 females)	16, 20, 24 °C	Rest, sleep	Gastrointestinal	−0.06 ± 0.26
	Januario et al. 2024 [106]	Healthy adults (33.4 ± 8.2 years)	15 (7 males, 8 females)	32 °C, 60% RH	Rest, exercise (cycling), recovery	Gastrointestinal	0.01 ± 0.18
	Kaltsatou et al. 2024 [107]	Chronic heart failure patients (53.5 ± 8.5 years)	12 (8 males, 4 females)	28 °C, 39% RH	Rest	Gastrointestinal	−0.14 ± 0.41
	Kaltsatou et al. 2024 [107]	Chronic heart failure patients (53.5 ± 8.5 years)	12 (8 males, 4 females)	28 °C, 39% RH	Exercise (Bruce protocol)	Gastrointestinal	0.06 ± 0.20
	Kaltsatou et al. 2024 [107]	Chronic heart failure patients (53.5 ± 8.5 years)	12 (8 males, 4 females)	28 °C, 39% RH	Recovery	Gastrointestinal	0.11 ± 0.14
	Ehler et al. 2025 [108]	ICU patients (63.3 ± 15.1 years)	112 (64 males, 48 females)	N/A	N/A	Bladder	−0.38 ± 0.43
	Quesada et al. 2025 [109]	Healthy adults (23 ± 4 years)	20 (10 males, 10 females)	22.2 ± 0.5 °C, 37 ± 10% RH	Rest, exercise (cycling), recovery	Gastrointestinal	−0.3 ± 0.2
	McLaughlin et al. 2025 [110]	Healthy adults (30.5 ± 9.23 years)	24 (13 males, 11 females)	35.9 ± 0.3 °C, 20.7 ± 3.3% RH	Exercise (cycling)	Rectal	0.15 ± 0.43
	Xu et al. 2025 [111]	Healthy males (24.7 ± 1.3 years)	24	40 °C, 57–58% RH, 0.15 ± 0.05 m/s	Free-living conditions ranging from rest to low-intensity daily activities (8 h)	Rectal	−0.19 ± 0.27
	Xu et al. 2025 [111]	Healthy females (23 ± 2 years)	14	40 °C, 57–58% RH, 0.15 ± 0.05 m/s	Free-living conditions ranging from rest to low-intensity daily activities (8 h)	Rectal	0.06 ± 0.26
	Xu et al. 2025 [111]	Healthy males (24.7 ± 1.3 years)	24	40 °C, 57–58% RH, 0.15 ± 3.2 ± 0.4 m/s	Free-living conditions ranging from rest to low-intensity daily activities (8 h)	Rectal	−0.34 ± 0.12
	Xu et al. 2025 [111]	Healthy females (23 ± 2 years)	14	40 °C, 57–58% RH, 0.15 ± 3.2 ± 0.4 m/s	Free-living conditions ranging from rest to low-intensity daily activities (8 h)	Rectal	−0.27 ± 0.10
Heat-flux method (DHF)	Feng et al. 2017 [115]	Healthy adults (26.8 ± 2.1 years)	34 (30 males, 4 females)	26 °C, 50–60% RH	Rest	Sublingual	0.13 ± 0.22
	Tokizawa et al. 2021 [119]	Healthy adults (37 ± 7 years)	21 (15 males, 6 females)	25 °C, 35 °C	Rest, exercise, recovery	Esophageal	0.00 ± 0.19
	Tokizawa et al. 2021 [119]	Healthy adult males (36 ± 8 years)	9	35 °C	Rest, exercise, recovery	Esophageal	−0.10 ± 0.23
	Tokizawa et al. 2021 [119]	Healthy adult males (36 ± 11 years)	11	30 °C	Rest, exercise, recovery	Esophageal	−0.09 ± 0.23
	Tokizawa et al. 2021 [119]	Healthy adult males (36 ± 11 years)	11	40 °C	Rest, exercise, recovery	Esophageal	−0.04 ± 0.18
	Tokizawa et al. 2021 [119]	Healthy adult males (30 ± 6 years)	8	35 °C	Rest, exercise, recovery	Esophageal	−0.01 ± 0.23
Heart rate (Kalman filter)	Buller et al. 2010 [124]	Soldiers, runners	25	20–40 °C	Exercise (low-to-moderate-intensity exercise, 2–8 h, intermittent)	Rectal, esophageal	0.30 ± 0.13
Heart rate (extended Kalman filter)	Buller et al. 2013 [127]	Healthy adults (20–30 years)	83 (82 males, 1 females)	9–45 °C, 9–97% RH, 0–4 m/s	Treadmill walking/running, cycling, long-distance marching/patrolling; ~1–24 h	Rectal, esophageal, gastrointestinal	−0.03 ± 0.32
	Looney et al. 2018 [132]	Healthy males (24 ± 3 years), and females (24 ± 4 years)	8 (6 males, 2 females)	18–22 °C	Sleep and light seated activities (≈16 h)	Gastrointestinal	0.00 ± 0.28
	Hunt et al. 2019 [125]	Healthy adult males (26.4 ± 6.0 years)	8	24 °C, 50% RH; 32 °C, 60% RH	Treadmill walking at 2.5–5.5 km/h with recovery periods	Gastrointestinal	0.01 ± 0.33
	Hagen et al. 2020 [134]	Healthy adult male athletes (American football)	13	N/A	Sports training (5 min × 18–22 sets)	Gastrointestinal	−0.11 ± 0.46
	Rizvi et al. 2022 [137]	Healthy 8 males (21.4 ± 2.3 years) and 8 males (22.7 ± 1.8 years)	16	36 ± 0.5 °C, 59 ± 5% RH, 0.17 ± 0.05 m/s	Treadmill walking at 4.5 km/h with recovery periods	Gastrointestinal	−0.03 ± 0.16
	de Korte et al. 2022 [140]	Elite male (26 ± 5) and female (26 ± 5) athletes	101 (49 males, 52 females)	31.6 ± 1.0 °C, 74 ± 5% RH	Graded exercise	Gastrointestinal	0.15 ± 0.23
	Peggen et al. 2024 [142]	General adults (some on cardiovascular/psychotropic medications) (56 ± 16)	18 (11 males, 7 females)	N/A	Free-living conditions ranging from rest to low-intensity daily activities (≈26 h)	Gastrointestinal	0.09 ± 0.22
Heart rate and other sensors	Niedermann et al. 2014 [10]	Healthy physically active males (23.0 ± 3.9 years)	10	30 ± 0.2 °C, 42.9 ± 1.1% RH, <0.3 m/s	Rest, exercise at 40% and 60% VO_2_max, recovery	Gastrointestinal	−0.17 ± 0.14
	Niedermann et al. 2014 [10]	Healthy physically active males (24.6 ± 2.0 years)	10	10.1 ± 0.2 °C, 49.5 ± 4.9% RH, 0.5 ± 0.1 m/s	Rest, exercise at 60% VO_2_max, recovery	Gastrointestinal	0.04 ± 0.28
	Welles et al. 2018 [133]	Healthy adult soldiers (22 ± 4 years)	8	25 °C, 50% RH; 35 °C, 70% RH; 40 °C, 20% RH	Rest, exercise, recovery	Gastrointestinal	−0.01 ± 0.09
	Moyen et al. 2021 [136]	Healthy adults (28.9 ± 7.8 years)	27 (19 males, 8 females)	13–43 °C, 11–75% RH	Rest, exercise, recovery	Rectal, gastrointestinal	0.07 ± 0.32
	Etienne et al. 2023 [141]	Post-vaccination healthy adults (35.8 ± 8.2 years)	17	N/A	Free-living conditions ranging from rest to low-intensity daily activities (≈26 h)	Gastrointestinal	0.11 ± 0.23

For studies reporting 95% confidence intervals, the corresponding standard deviations were estimated by dividing the CI width by 1.96. N/A: not available.

## Data Availability

The author confirm that the data supporting the findings of this study are available within the article.
